# 4-Amino­pyridinium azide 4-amino­pyridine solvate

**DOI:** 10.1107/S1600536810044843

**Published:** 2010-11-06

**Authors:** Hui-Fen Qian, Wei Huang

**Affiliations:** a College of Sciences, Nanjing University of Technology, Nanjing 210009, People’s Republic of China; b State Key Laboratory of Coordination Chemistry, Nanjing National Laboratory of Microstructures, School of Chemistry and Chemical Engineering, Nanjing University, Nanjing 210093, People’s Republic of China

## Abstract

In the title compound, C_5_H_7_N_2_^+^·N_3_^−^·C_5_H_6_N_2_, all N atoms of the azide anion are situated on a twofold rotational axis, so the 4-amino­pyridinium cation and 4-amino­pyridine mol­ecule, being related by symmetry, occupy one position in the asymmetric unit. Inter­molecular N—H⋯N hydrogen bonds generate a three-dimensional hydrogen-bonding network which consolidates the crystal packing.

## Related literature

For a related compound, see: Teulon *et al.* (1985 ▶).
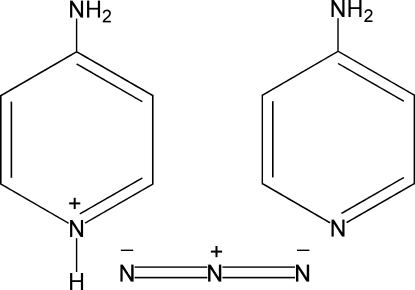


## Experimental

### 

#### Crystal data

C_5_H_7_N_2_^+^·N_3_^−^·C_5_H_6_N_2_
*M* *_r_* = 231.27
Monoclinic, 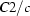

*a* = 7.507 (3) Å
*b* = 12.247 (5) Å
*c* = 13.634 (5) Å
β = 99.278 (5)°
*V* = 1237.0 (8) Å^3^
*Z* = 4
Mo *K*α radiation
μ = 0.08 mm^−1^
*T* = 291 K
0.14 × 0.11 × 0.10 mm

#### Data collection

Bruker SMART 1K CCD area-detector diffractometer
Absorption correction: multi-scan (*SADABS*; Bruker, 2000 ▶) *T*_min_ = 0.988, *T*_max_ = 0.992
3027 measured reflections
1096 independent reflections
852 reflections with *I* > 2σ(*I*)
*R*_int_ = 0.072

#### Refinement

*R*[*F* ^2^ > 2σ(*F* ^2^)] = 0.036
*wR*(*F* ^2^) = 0.105
*S* = 1.08
1096 reflections
80 parameters
1 restraint
H-atom parameters constrained
Δρ_max_ = 0.11 e Å^−3^
Δρ_min_ = −0.11 e Å^−3^

### 

Data collection: *SMART* (Bruker, 2000 ▶); cell refinement: *SAINT* (Bruker, 2000 ▶); data reduction: *SAINT*; program(s) used to solve structure: *SHELXTL* (Sheldrick, 2008 ▶); program(s) used to refine structure: *SHELXTL*; molecular graphics: *SHELXTL*; software used to prepare material for publication: *SHELXTL*.

## Supplementary Material

Crystal structure: contains datablocks global, I. DOI: 10.1107/S1600536810044843/cv2787sup1.cif

Structure factors: contains datablocks I. DOI: 10.1107/S1600536810044843/cv2787Isup2.hkl

Additional supplementary materials:  crystallographic information; 3D view; checkCIF report

## Figures and Tables

**Table 1:** Hydrogen-bond geometry (Å, °)

*D*—H⋯*A*	*D*—H	H⋯*A*	*D*⋯*A*	*D*—H⋯*A*
N1—H1*A*⋯N5^i^	0.86	2.15	3.008 (2)	174
N1—H1*B*⋯N3^ii^	0.86	2.14	2.9942 (18)	172
N2—H2*A*⋯N2^iii^	0.86	1.84	2.689 (3)	169

Symmetry codes: (i) 
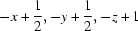
; (ii) 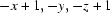; (iii) 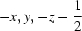.

**Fig. 1.**
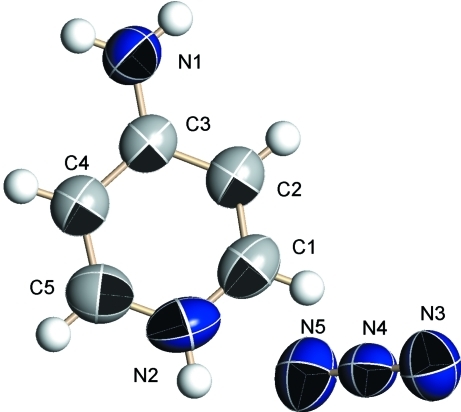
Molecular structure of the title compound with the atom-numbering scheme. Displacement ellipsoids are drawn at the 30% probability level.

## supplementary crystallographic information

### Comment

The crystal structure of 4-aminopyridine hemiperchlorate, has been previously reported (Teulon *et al.*, 1985). In this paper, we report the X-ray single-crystal structure of 4-aminopyridinium azide 4-aminopyridine (I).

The molecular structure of (I) is illustrated in Fig. 1. All N atoms of the azide anions are situated on a twofold rotational axis, so 4-aminopyridinium cation and 4-aminopyridine molecule being related by symmetry occupy one position in the asymmetric unit. Intermolecular N—H···N hydrogen bonds (Table 1) generate a three-dimensional hydrogen-bonding network which consolidate the crystal packing.

### Experimental

The title compound (I) was prepared by the treatment of 4-aminopyridine (0.5 mmol, 0.041 g) and excess sodium azide (NaN_3_) in 20 ml methanol with a few drops of acetate acid (HOAc). Colourless single crystals suitable for X-ray diffraction measurement were grown from its methanol solution after five days' slow evaporation at room temperature in air. Anal. Calcd. for C_10_H_13_N_7_: C, 51.94; H, 5.66; N, 42.40%. Found: C, 51.85; H, 5.81; N, 42.29%. FT–IR (KBr pellets, cm^-1^): 3447 (*vs*), 2057 (*s*), 1645 (*s*), 1463 (*m*), 1202 (w), 1202 (w), 840 (w), and 590 (w).

### Refinement

One restraint (DELU 0.001 C1 C2) was used to reduce the components of the anisotropic displacement parameters along chemical C—C bond. The H atoms were placed in geometrically idealized positions and refined as riding, with C—H = 0.93 Å and N—H = 0.86 Å, *U*_iso_(H) = 1.2*U*_eq_(C) and *U*_iso_(H) = 1.2*U*_eq_(N).

### Crystal data

**Table:**

C_5_H_7_N_2_^+^·N_3_^−^·C_5_H_6_N_2_	*F*(000) = 488
*M**_r_* = 231.27	*D*_x_ = 1.242 Mg m^−^^3^
Monoclinic, *C*2/*c*	Mo *K*α radiation, λ = 0.71073 Å
Hall symbol: -C 2yc	Cell parameters from 1359 reflections
*a* = 7.507 (3) Å	θ = 3.0–25.4°
*b* = 12.247 (5) Å	µ = 0.08 mm^−^^1^
*c* = 13.634 (5) Å	*T* = 291 K
β = 99.278 (5)°	Block, colourless
*V* = 1237.0 (8) Å^3^	0.14 × 0.11 × 0.10 mm
*Z* = 4	

### Data collection

**Table:**

Bruker SMART 1K CCD area-detector diffractometer	1096 independent reflections
Radiation source: fine-focus sealed tube	852 reflections with *I* > 2σ(*I*)
graphite	*R*_int_ = 0.072
φ and ω scans	θ_max_ = 25.0°, θ_min_ = 3.0°
Absorption correction: multi-scan (*SADABS*; Bruker, 2000)	*h* = −8→8
*T*_min_ = 0.988, *T*_max_ = 0.992	*k* = −12→14
3027 measured reflections	*l* = −16→15

### Refinement

**Table:**

Refinement on *F*^2^	Secondary atom site location: difference Fourier map
Least-squares matrix: full	Hydrogen site location: inferred from neighbouring sites
*R*[*F*^2^ > 2σ(*F*^2^)] = 0.036	H-atom parameters constrained
*wR*(*F*^2^) = 0.105	*w* = 1/[σ^2^(*F*_o_^2^) + (0.0493*P*)^2^ + 0.0478*P*] where *P* = (*F*_o_^2^ + 2*F*_c_^2^)/3
*S* = 1.08	(Δ/σ)_max_ < 0.001
1096 reflections	Δρ_max_ = 0.11 e Å^−^^3^
80 parameters	Δρ_min_ = −0.11 e Å^−^^3^
1 restraint	Extinction correction: *SHELXTL* (Sheldrick, 2008), Fc^*^=kFc[1+0.001xFc^2^λ^3^/sin(2θ)]^-1/4^
Primary atom site location: structure-invariant direct methods	Extinction coefficient: 0.042 (5)

### Special details

**Table:**

Experimental. The structure was solved by direct methods (Bruker, 2000) and successive difference Fourier syntheses.
Geometry. All e.s.d.'s (except the e.s.d. in the dihedral angle between two l.s. planes) are estimated using the full covariance matrix. The cell e.s.d.'s are taken into account individually in the estimation of e.s.d.'s in distances, angles and torsion angles; correlations between e.s.d.'s in cell parameters are only used when they are defined by crystal symmetry. An approximate (isotropic) treatment of cell e.s.d.'s is used for estimating e.s.d.'s involving l.s. planes.
Refinement. Refinement of *F*^2^ against ALL reflections. The weighted *R*-factor *wR* and goodness of fit *S* are based on *F*^2^, conventional *R*-factors *R* are based on *F*, with *F* set to zero for negative *F*^2^. The threshold expression of *F*^2^ > σ(*F*^2^) is used only for calculating *R*-factors(gt) *etc*. and is not relevant to the choice of reflections for refinement. *R*-factors based on *F*^2^ are statistically about twice as large as those based on *F*, and *R*-factors based on ALL data will be even larger.

### Fractional atomic coordinates and isotropic or equivalent isotropic displacement parameters (Å^2^)

**Table:**

	*x*	*y*	*z*	*U*_iso_*/*U*_eq_	Occ. (<1)
C1	0.1721 (2)	0.05406 (12)	−0.09812 (12)	0.0879 (5)	
H1	0.2321	0.0033	−0.1316	0.106*	
C2	0.22248 (18)	0.06466 (10)	0.00146 (11)	0.0780 (4)	
H2	0.3145	0.0214	0.0350	0.094*	
C3	0.13523 (16)	0.14103 (10)	0.05345 (9)	0.0699 (4)	
C4	−0.00162 (18)	0.20282 (11)	−0.00180 (11)	0.0791 (4)	
H4	−0.0635	0.2547	0.0294	0.095*	
C5	−0.0439 (2)	0.18672 (13)	−0.10131 (12)	0.0932 (5)	
H5	−0.1355	0.2286	−0.1371	0.112*	
N1	0.18164 (16)	0.15416 (9)	0.15211 (9)	0.0858 (4)	
H1A	0.1265	0.2016	0.1828	0.103*	
H1B	0.2665	0.1151	0.1846	0.103*	
N2	0.04055 (19)	0.11306 (11)	−0.15027 (9)	0.0948 (4)	
H2A	0.0108	0.1042	−0.2134	0.114*	0.50
N3	0.5000	−0.02503 (17)	0.7500	0.1009 (6)	
N4	0.5000	0.07152 (17)	0.7500	0.0760 (5)	
N5	0.5000	0.16656 (17)	0.7500	0.1062 (6)	

### Atomic displacement parameters (Å^2^)

**Table:**

	*U*^11^	*U*^22^	*U*^33^	*U*^12^	*U*^13^	*U*^23^
C1	0.0948 (10)	0.0867 (9)	0.0891 (8)	−0.0181 (8)	0.0353 (8)	−0.0118 (8)
C2	0.0744 (8)	0.0752 (8)	0.0879 (8)	−0.0108 (6)	0.0231 (6)	−0.0057 (6)
C3	0.0681 (7)	0.0682 (7)	0.0762 (9)	−0.0163 (6)	0.0206 (6)	−0.0037 (6)
C4	0.0765 (8)	0.0799 (8)	0.0834 (9)	−0.0071 (6)	0.0205 (7)	−0.0009 (7)
C5	0.0938 (10)	0.1000 (11)	0.0854 (11)	−0.0100 (8)	0.0132 (8)	0.0090 (8)
N1	0.0900 (8)	0.0873 (8)	0.0803 (8)	0.0016 (5)	0.0144 (6)	−0.0086 (6)
N2	0.1105 (10)	0.1050 (9)	0.0714 (8)	−0.0251 (7)	0.0223 (7)	−0.0032 (7)
N3	0.0964 (13)	0.0868 (12)	0.1185 (15)	0.000	0.0141 (10)	0.000
N4	0.0626 (9)	0.0993 (13)	0.0671 (9)	0.000	0.0139 (6)	0.000
N5	0.1123 (14)	0.0918 (14)	0.1242 (16)	0.000	0.0488 (12)	0.000

### Geometric parameters (Å, °)

**Table:**

C1—N2	1.333 (2)	C4—H4	0.9300
C1—C2	1.356 (2)	C5—N2	1.341 (2)
C1—H1	0.9300	C5—H5	0.9300
C2—C3	1.3985 (18)	N1—H1A	0.8600
C2—H2	0.9300	N1—H1B	0.8600
C3—N1	1.3437 (17)	N2—H2A	0.8600
C3—C4	1.395 (2)	N3—N4	1.182 (3)
C4—C5	1.357 (2)	N4—N5	1.164 (2)
			
N2—C1—C2	123.06 (14)	C3—C4—H4	120.2
N2—C1—H1	118.5	N2—C5—C4	122.83 (15)
C2—C1—H1	118.5	N2—C5—H5	118.6
C1—C2—C3	119.58 (14)	C4—C5—H5	118.6
C1—C2—H2	120.2	C3—N1—H1A	120.0
C3—C2—H2	120.2	C3—N1—H1B	120.0
N1—C3—C4	121.68 (12)	H1A—N1—H1B	120.0
N1—C3—C2	121.36 (13)	C1—N2—C5	117.93 (13)
C4—C3—C2	116.97 (13)	C1—N2—H2A	121.0
C5—C4—C3	119.63 (14)	C5—N2—H2A	121.0
C5—C4—H4	120.2	N5—N4—N3	180.000 (1)
			
N2—C1—C2—C3	−0.5 (2)	C2—C3—C4—C5	0.10 (18)
C1—C2—C3—N1	−179.81 (11)	C3—C4—C5—N2	0.0 (2)
C1—C2—C3—C4	0.11 (17)	C2—C1—N2—C5	0.6 (2)
N1—C3—C4—C5	−179.98 (11)	C4—C5—N2—C1	−0.3 (2)

### Hydrogen-bond geometry (Å, °)

**Table:**

*D*—H···*A*	*D*—H	H···*A*	*D*···*A*	*D*—H···*A*
N1—H1A···N5^i^	0.86	2.15	3.008 (2)	174
N1—H1B···N3^ii^	0.86	2.14	2.9942 (18)	172
N2—H2A···N2^iii^	0.86	1.84	2.689 (3)	169

Symmetry codes: (i) −*x*+1/2, −*y*+1/2, −*z*+1; (ii) −*x*+1, −*y*, −*z*+1; (iii) −*x*, *y*, −*z*−1/2.
